# Non Insulin Dependent Diabetes in Sand Rat (*Psammomys obesus*) and Production of Collagen in Cultured Aortic Smooth Muscle Cells. Influence of Insulin

**DOI:** 10.1155/EDR.2001.37

**Published:** 2001

**Authors:** S. Aouichat Bouguerra, M. C. Bourdillon, Y. Dahmani, F. Bekkhoucha

**Affiliations:** 1 Laboratory of Nutrition and Metabolism Physiology P Box 32 ISN, USTHB Algiers Algeria; 2 lNSERM U 63 22 Avenue du Doyen Lepine Case 18 Lyon Bron Cedex 69675 France; 3 Central Hospital of Army Ain Naadja Algiers Algeria

## Abstract

In this report, we have shown that the standard laboratory diet administered to *Psammomys obesus* (sand rat) from Beni Abbes in Algeria, induced a non-insulin dependant diabetes, characterised by increase of body weight (p<0.001) as well as hyperinsulinemia, hyperglycemia and hypercholesterolemia. In cultured aortic smooth muscle cells (SMC) of sand rats, type I and type III collagen biosynthesis and insulin effects, at low dose, on these parameters were investigated. In all experimental conditions of cultured SMC study, The α chains of type I collagen were analysed by immunoblotting in media and cells.

Metabolic radiolabelling and Immunochemical procedures revealed that, in diabetic state, synthetic SMC (SMCs) actively produce type I and III collagen which are synthesised in the cells and secreted in the medium; type I collagen was predominant as compared with type III collagen. Diabetes enhanced the collagen synthesis. Low dose of Insulin added to the medium, during 48h of incubation, induced a marked reduction in the synthesis of collagen types, especially type I collagen.


*Int. Jnl. Experimental Diab. Res.*, Vol. 2, pp. 37–46
Reprints available directly from the publisher
Photocopying permitted by license only

© 2001 OPA (Overseas Publishers Association) N.V.
Published by license under
the Harwood Academic Publishers imprint,
part of Gordon and Breach Publishing,
a member of the Taylor & Francis Group.
Printed in the United States.

### Non Insulin Dependent Diabetes in Sand Rat (*Psammomys obesus*) and Production of Collagen in Cultured Aortic Smooth Muscle Cells. Influence of Insulin

S. AOUICHAT BOUGUERRA[a,*], M. C. BOURDILLON[c], Y. DAHMANI[a] and F. BEKKHOUCHA[b]

[a]*Laboratory of Nutrition and Metabolism Physiology, P Box 32, ISN, USTHB, Algiers;*
[b]*INSERM U 63, 22 Avenue du Doyen Lepine, Case 18, 69675 Lyon Bron Cedex, France;*
[c]*Central Hospital of Army, Ain Naadja, Algiers*

*(Received 23 May 2000; In final form 19 December 2000)*

**In this report, we have shown that the standard laboratory diet administered to *Psammomys obesus* (sand rat) from Beni Abbes in Algeria, induced a non-insulin dependant diabetes, characterised by increase of body weight ($p<0.001$) as well as hyperinsulinemia, hyperglycemia and hypercholesterolemia. In cultured aortic smooth muscle cells (SMC) of sand rats, type I and type III collagen biosynthesis and insulin effects, at low dose, on these parameters were investigated. In all experimental conditions of cultured SMC study, The α chains of type I collagen were analysed by immunoblotting in media and cells.**

**Metabolic radiolabelling and Immunochemical procedures revealed that, in diabetic state, synthetic SMC (SMCs) actively produce type I and III collagen which are synthesised in the cells and secreted in the medium; type I collagen was predominant as compared with type III collagen. Diabetes enhanced the collagen synthesis. Low dose of Insulin added to the medium, during 48h of incubation, induced a marked reduction in the synthesis of collagen types, especially type I collagen.**

*Keywords*: Sand rat; Non-insulin dependent diabetes; Smooth muscle cells; Type I and type III collagen; Insulin

*Corresponding author.

### INTRODUCTION

The atherosclerotic process affects the intima and media; it concerns all the cells of the aortic wall especially smooth muscle cells (SMC).[1] The migration of SMC from media to intima and the change of the SMC phenotype from contractile state to synthetic state are considered as the major events in the atherosclerotic process.[2,3] They are caused by endothelial cell dysfunctioning[1,4] which is signalled to SMC by cell to cell interaction.[5,6] Thus, SMC undergo the change of their phenotype and become synthetic and proliferative (SMCs).[7] Cultured SMCs show analogies with the observed SMCs in atherosclerotic lesions; they spontaneously lose their contraction capacities and increase their secretion capacities.[8] Similar changes occur in non-insulin dependent diabetic patients, which are characterised by hyperinsulinemia, insulin-resistance, increased LDL and decreased HDL [9,10] representing greater cardiovascular risks.

During diabetes, atherosclerosis process is accelerated. The increased SMC proliferation is an abnormality, which aggravates the process [11] as well as the dysfunctioning of the extracellular matrix (ECM) especially the quan- titative changes of the collagen. In fact, the SMC is engaged in repetitive divisions and in an ECM synthesis in non-conformity to the genetic coded program at a contractile. [12,13]

Our study was carried out on aortic SMCs cultured from diabetic *Psammomys obesus*. This model has the characteristic of developing a nutritional diabetic syndrome comparable to clinical diabetes with degenerative and vascular complications. [14] This study involves morphometric characteristics of *Psammomys obesus* cultured SMCs, in different experimental conditions as well as biosynthesis of type I and III collagen and immunological and the identification of α chains. Insulin influence at low dose on the SMCs of diabetic *Psammomys obesus* was also assessed.

### MATERIALS AND METHODS

### Animals

Our study was carried out on 14 sand rats (*Psammomys obesus*). This animal is native of Beni Abbes region (wilaya of Béchar, southwest of Algeria, 30°7 latitude north and 2°10 longitude west). It is a species with daytime activity and belongs to the *Muridae* family and *Gerbillidae* subfamily. It has a life expectancy of 3 years and in his biotope eats almost exclusively halophile plants (rich in water and minerals) belonging to the *Chenopodiaceae* family such as *Traganum nudatum*, *Suaeda mollis* and *Salsola foetida*. [15]

When trapped, animals were allowed to adapt to laboratory conditions for 15 days and fed on natural vegetables only. In the animal room, animals were maintained in conditions previously described by Marquié *et al.* [16]

Animals of matched ages (2–3 months) and adapted to laboratory conditions were divided into two groups: 7 were fed a natural plant diet (50g/day) during 6 months and the daily caloric intake was 20kcal. 7 animals were fed a laboratory diet (rich in carbohydrates) with salt water *"ad libitum"* (NaCl 0.9%) during the same period. Their food consumption (10g/day) represented a higher caloric intake (32.5kcal/day/animal). At the time of killing, sand rats were 9 months old.

Animals fed on high caloric diet showed a relative weight gain and insulin levels increased over the weeks that followed. After the third month, they developed a non insulin dependent diabetes as described by Marquié *et al.*[16] At the sixth month of experiments, animals of the two groups: the normal group placed on hypocaloric diet (control group) and the diabetic group feeding high caloric diet, were killed.

### Analytical Techniques

The animals were bled from the retro-orbital venous plexus; this technique eliminates using anaesthesic agents which affect measurements of biochemical parameters. Blood collected in tubes containing heparin was centrifuged at 3000 rpm for 10 min and plasma was stored at - 30°C.

Blood glucose and cholesterol were measured by the enzymatic colorimetric method using a test kit of Boehringer. Blood Insulin was determined by radioimmunoassay using CIS test kit (ORIS INDUS).

### Aortic SMC Culture

Cellular culture technique was used according to Ross,[17] modified by Bourdillon *et al.*[18] Figure 1. For cultured SMC, explants were obtained from normal and diabetic thoracic aorta. They were prepared after removing

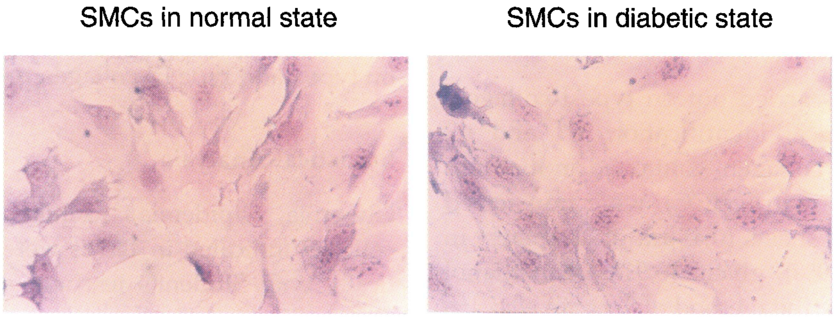

FIGURE 1 Microscopic appearance of cultured aortic SMC at synthetic phenotype, they were fixed in aqueous bath and stained in May Grunwald Giemsa. X 200. In proliferative phase, aortic SMC of normal and diabetic *Psammomys obesus* were in synthetic state and presented a polygonal aspect.

adventitia collagenage action at 0,1% (type IA, Sigma, USA) and incu-bated in DMEM (Gibco, USA), supplemented with 10% foetal calf serum (SIGMA, USA), penicillin (50UI/ml), streptomycin (5μg/ml) and glutamine at 200mM (Gibco, USA). The explants are maintained at 37°C under air: $CO_2$ (95% : 5%) atmosphere until they reached confluence. Then, they were trypsinized and subcultured. For the experiments, synthetic cells (SMCs) were used in the fourth passage of proliferative phase ($10^6$ cells/ml).

### Morphometric Measurements

In proliferative state, the medium was eliminated from the patches and SMCs were washed with PBS at 10% then fixed in aqueous bath and coloured with May Grunwald Giemsa. Morphometric analysis was carried out of on 100 measurements of cellular and nuclear great axes as well as nucleolus numeration.

### Collagen Biosynthesis

According to the method of Peterkofsky *et al.*, [19] cells were labeled with L-5-$^3$H proline (10μCi/ml, specific activity 20Ci/mmol, Isotopchim) during 24h in medium culture without foetal calf serum containing 10μg/ml of ascorbic acid. Total collagen, in medium and cells, was estimated after three successive dialyses; the first two against water, the third against acetic acid 0,5M and pepsin (200μg/ml).

Radioactivity was measured by liquid scintillation and results were expressed in cpm/$10^6$cells. The collagen biosynthesis relative to total protein synthesis was estimated according to Wiestner *et al.* [20]

### Collagen Types

After pepsination, medium (ECC) and cells (ICC) were lyophilised and resuspended in buffer solution (tris HCl, 0.05M, SDS 14%, bromophenol blue 0,05%, glycerol 0,5% and EDTA 2mM). The different fractions of radiolabeled collagen chains were separated by vertical electrophoresis on polyacrylamide gel (SDS-PAGE) according to the procedure of Laemmli.[21] After the gel discolouration, $\alpha 1$ and $\alpha 2$ collagenous chains were evaluated by densitometric analysis. At the end of this step, the gel was dried for 3h at 80°C and the $\alpha$ chains of collagen separated on SDS-PAGE were quantified by excision of each $\alpha$ band. The radioactivity was eluted in hydrogen peroxide at 12% during 24h. The radioactivity was measured by liquid scintillation and expressed in cpm/$10^6$ cells. The radioactivity recovered in type I and type III collagen was quantified by numeric integration.

### Immunoblotting

This study was carried out on type I collagen. For immunological identification, α1 and α2 chains of type I collagen, contained in medium (ECC) and cells matrix (ICC), were transferred to nitrocellulose sheets (0,45μm of diameter) in tris buffer (0,189%), glycin (0,912%), methanol 20% at pH 8,6 by the method of Towbin *et al.* [22] Transferred proteins were incubated with first antiserum (at 4/100 dilution). After an overnight incubation at 4°C, a second specific antiserum (at 1/250 dilution, Sanofy) linked to the peroxidase was added to bind the antigen-antibody complex during a 2-h period at room temperature; the bands were revealed by tetrachloride 3-3 DAB 0.5%.

We prepared in our laboratory the rabbit antiserum against bovine type I collagen (Sigma) and the goat antiserum against bovine type III collagen (Sigma) used in the immunological procedures.

Antisera were titrated by qualitative antigen diffusion (type I or III collagen) and corresponding antiserum by distribution in wells bored in 1.5% agar gel. The antigen–antibody reaction was accelerated under low voltage (25A, 150 V) during 3h.

### Influence of Insulin

Human insulin (Mixtar) was added at low doses (4mU/ml) to the medium of diabetic *Psammomys obesus* SMCs, during 48-h, to analyse his effects on morphological variation cells and on type I and type III collagen production.

### Statistical Analysis

Glycemia, lipidemia, cellular proteins and collagen indicated in Tables were analysed statistically with Student's test and are expressed as mean ± SD; insulin was analysed with Mann Whitney test.

### RESULTS

### Biochemical Study

The biochemical parameters can be seen in Table I. *Psammomys obesus* on the high caloric diet became diabetic, exhibited hyperglycemia and hypercholesterolemia with increase of esterified and free cholesterol compared with the control group (natural diet). These metabolic changes were associated with excess body weight ($p<0.001$).

### Morphometric Study

Morphometric results indicated in the Table II showed that cellular (LC) and nuclear (LN) great axes were increased in the diabetic state ($p<0{,}0001$ for LC ; $p<0.001$ for LN). The number of nucleoli augmented from 3.2 ± 0.9 in control group to 4.9 ± 1.3 in diabetic group. The insulin influence during 48h caused a slight decrease of

TABLE I Evolution of body weight and plasma biochemical parameters in *Psammomys obesus* became diabetic under hypercaloric diet during 6 months. Animals were killed at 9 months old

| Captivity time | 0 Month | 6 Months | | | |
|---|---|---|---|---|---|
| Food in captivity | Control fed on natural diet | Control fed on natural diet | | Diabetic fed on hypercaloric diet | |
| Body weight(g) | 77,7 ± 4,4 | 92,2 ± 3,9 | $p<0{,}02$ | 114,7 ± 9,6 | $p<0{,}001$ |
| Glucose (mg/100ml) | 60,2 ± 5,1 | 69,0 ± 7,0 | $p<0{,}02$ | 213,5 ± 21,2 | $p<10^{-5}$ |
| Insulin(μU/ml) | 25,2 ± 5,9 | 37,3 ± 7,1 | $p<0{,}05$ | 222,0 ± 26,1 | $p<10^{-5}$ |
| Esterified cholesterol (mg/100ml) | 34,3 ± 4,7 | 45,2 ± 5,5 | $p<0{,}04$ | 71,3 ± 8,9 | $p<3\times10^{-4}$ |
| Free cholesterol (mg/100ml) | 17,4 ± 2,8 | 22,8 ± 2,7 | $p<0{,}05$ | 38,5 ± 7,2 | $p<0{,}001$ |

Values are means ± SD of 7 animals in control group and 7 animals in diabetic group.

TABLE II Synthetic SMC morphometric characteristics of *Psammomys obesus* (P.ob) in different experimental conditions (Normal (N), Diabetic (D), Diabetic + Insulin (D+1))

| | SMCs | | | | |
|---|---|---|---|---|---|
| | Normal P.ob | Diabetic P.ob | | Diabetic P.ob + Ins | |
| Great cell axes (μm) | 47,2±5,2 | 61,9±5,4 | $p<0,0001$ | 56,2±6,4 | $p<0,0001$ |
| Great nuclear axes(μm) | 21,6±1,9 | 30,8±3,4 | $p<0,001$ | 26,4±3,5 | $p<0,001$ |
| Nucleolus number | 3,2±0,9 | 4,9±1,3 | $p<0,0001$ | 3,7±0,8 | $p<0,001$ |

Values are means±SD of 100 determinations for each experimental group. Significance tests refer to control group.

the morphometric parameters and nucleoli number (p<0,0001 for LC; p<0.001 for LN and nucleoli number).

### Biosynthesis of Total Collagen

The results of these parameters were indicated in Table III and Figure 2. Normal *Psammomys obesus* SMCs presented a total collagen synthesis equal to 1031±69 cpm/$10^6$ cells; the incorporation was statistically in favour of the medium rather than cells (p<0,0001). The rate of total collagen and secretion were respectively equal to 39.5±3.3% and 46.6±1.8%. Diabetic *Psammomys obesus* SMCs showed an increased total collagen synthesis. The labelled proline incorporation remained more marked in the ECC (4060±160 *vs* 2892±242 cpm/$10^6$ cells in the ICC; p<0,0005) and the secretion reached 86.7±9.4%.

When insulin was added to the medium of diabetic *Psammomys obesus* SMCs, the two compartments (medium and cells) showed a pronounced decrease (p<0,0001) but comparative to the basal state, they remain statistically higher (p<0,0001).

### Effect of Diabetes on Type I and Type III Collagen Synthesis

The results of [$^3$H] proline incorporation in type I and III collagen are shown in Table IV, Figures 3 and 4. In the two compartments of normal *Psammomys obesus* SMCs, type I collagen was more abundant than type III collagen (p<0,005 for medium, p<0,004 for cells). In addition, type I and III collagen were higher in the medium compared to cells. In fact results indicated respectively 254±24 and 175±34 cpm/$10^6$ cells in the medium; 115±10 and 72±7 cpm/$10^6$ cells

TABLE III Total collagen synthesis (cpm/$10^6$cells), secretion of total collagen (%) in cultured SMCs of P. ob in the three experimental conditions studied

| | SMCs | | | | |
|---|---|---|---|---|---|
| | Normal p.ob | Diabetic P.ob | | Diabetic P.ob + Ins | |
| ECC (cpm/$10^6$cells) | 720±72 | 4060±170 | $p<2\times10^{-8}$ | 1197±61 | $p<0,0001$ |
| ICC (cpm/$10^6$cells) | 311±26 | 2891±234 | $p<4\times10^{-6}$ | 1179±114 | $p<10^{-5}$ |
| ($^3$H) prolin incorporation in total collagen (cpm/$10^6$cells) | 1031±69 | 6951±1184 | $p<2\times10^{-9}$ | 2374±120 | $p<10^{-6}$ |
| Total collagen synthesis (%) | 39,5±3,3 | 148,5±10,3 | $p<2\times10^{-5}$ | 79,7±5,8 | $p<0,0001$ |
| Secretion (%) | 46,6±1,8 | 86,7±9,4 | $p<10^{-6}$ | 44,6±8,5 | ns |

Values are means±SD of six determinations and significance test refer to control group. Radiolabeling in total collagen is determined by total pepsin resistant radiolabelling in medium and cells.

Total collagen synthesis (%): Total pepsin resistant radiolabelling in medium+cells/Total protein radiolabelling in medium+cells Collagen secretion (%): Pepsin resistant radiolabelling in medium/Total pepsin resistant radiolabelling in medium+cells.

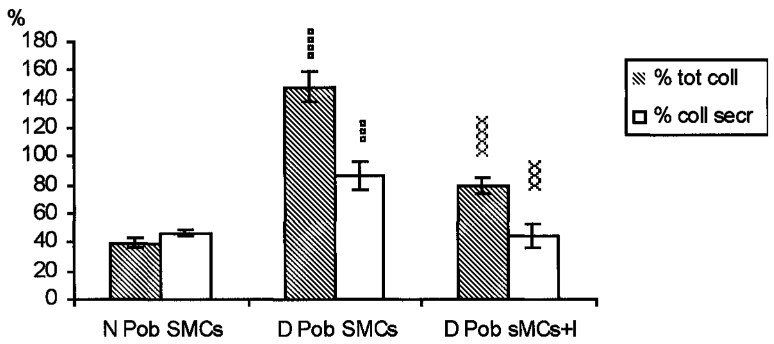

FIGURE 2 Total collagen synthesis (tot coll) and secretion (tot secr) by cultured SMCs of P.ob in the three experimental groups. (°) significance test between diabetic state and basal state. (x) significance test between diabetic state and insulin influence. Data are mean ± SD of six determinations.

TABLE IV ($^3$H) Proline incorporation in type I and type III collagen by cultured SMCs of P.ob in different experimental conditions. Collagen types were separated on SDS-PAGE electrophoresis and incorporation of ($^3$H) Proline in the two compartments (medium and cells) was determinated as described in Materials and Methods

| | | SMCs | | | | | |
|---|---|---|---|---|---|---|---|
| | | Normal P.ob | | Diabetic P.ob | | Diabetic P.ob + Ins | |
| Extracellular compartment | Collagen I | 254 ± 24 | $p < 0{,}0001$ | 2351 ± 116 | $p < 0{,}0001$ | 510 ± 25 | $p < 0{,}01$ |
| (cpm/10$^6$cells) | Collagen III | 175 ± 34 | | 1410 ± 83 | | 447 ± 31 | |
| Intracellular compartment | Collagen I | 115 ± 10 | $p < 0{,}004$ | 1503 ± 143 | $p < 10^{-5}$ | 541 ± 46 | $p < 0{,}01$ |
| (cpm/10$^6$cells) | Collagen III | 72 ± 07 | | 834 ± 67 | | 415 ± 64 | |

Values are means ± SD of six determinations, significance test compare type I collagen to type III collagen.

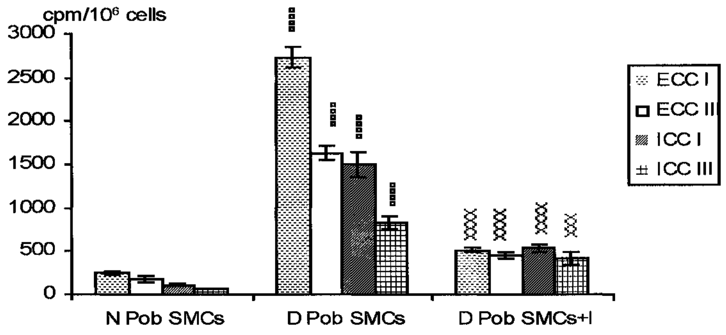

FIGURE 3 [$^3$H] Pro incorporation into type I and type III collagen in medium (ECC) and cells (ICC). In the three experimental groups (Normal, Diabetic, Diabetic 1 Insulin), SMC were at synthetic state. (°) Significance test between diabetic state and basal state. (x) Significance test between insulin influence and diabetic state. Data are mean ± SD of six determinations.

(p<0,0001 for type I collagen, p<0,0002 for type III collagen). Total type I and III collagen was estimated respectively as 56.5±3.2% and 36.0± 2.3%.

In diabetic *Psammomys obesus* SMCs, type I and type III collagen were statistically very elevated in the two compartments but remained pre-dominant in the medium. Thus, we have noted respectively 2351±116 and 1410±83 cpm/10$^6$ cells *vs* 1503±143 and 834±67cpm/10$^6$ cells in cells.

Besides, in both compartments, type I collagen presented a higher increase as compared to type III collagen. In fact, in the medium type I collagen

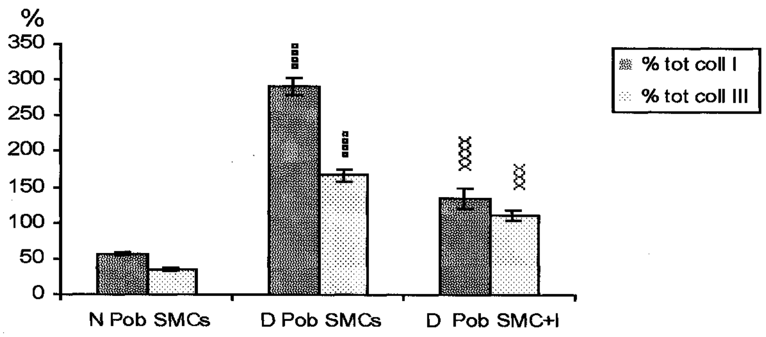

FIGURE 4 Total type I and type III collagen synthesis of P.ob cultured in the three experimental groups, determined by numeric integration from collagen types in medium and cells. (°) Significance test between diabetic state and basal state. (x) Significance test between insulin influence and diabetic state. Data are mean ± SD of six determinations.

Immunological procedures

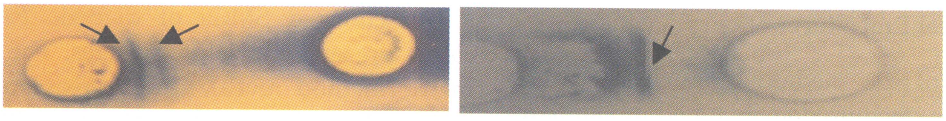

FIGURE 5 Immunodiffusion test of anti rabbit type I and anti goat type III collagen serum with corresponding antigens (type I and type III collagen from Sigma). In Figure 5a two specific arc corresponding to anti goat type III collagen serum (anti α1 and anti α2 chains serum), in Figure 5b one specific arc corresponding to anti goat type III collagen serum (anti α1 chains serum).

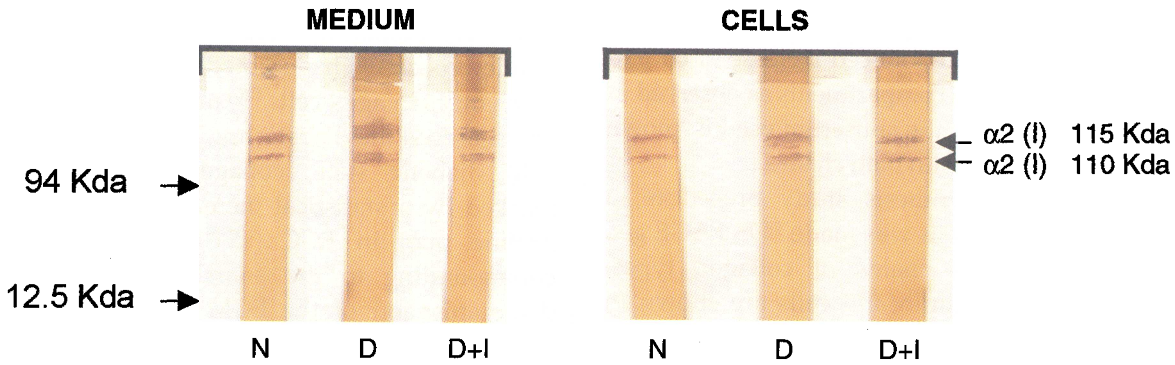

FIGURE 6 Immunological indentification of type I collagen in medium and cells of cultured SMCs in three experiments conditions normal (N), diabetic (D), diabetic + insulin (D+1) on the production of this protein. Equal aliquot of the latter media and the cell-matrix extract from aortic SMCs were submitted to SDS PAGE electrophoresis and immunoblotted with anti-rabbit type I collagen serum (at 4/100 dilution). Bound antibody was detected with a second specific antiserum: anti-anti-rabbit serum (at 1/250 dilution) linked to the peroxidase and visualised by tetrachloride 3-3 DAB 0.5%. Designated markers were sized between phosphorylase (94 Kda) and cytochrome C (12.5 Kda).

increased in the ratio of 9,3 *versus* 8,1 for type III collagen. In cells, the respective increase ratios of type I and type III collagen were 13.1 and 11.6.

The insulin influence, during 48-h at low dose (4 mU/ml) has induced a strong decrease of the radioproline incorporated into both compart-ments ($p<0.0001$). However, this decrease was more pronounced for type I collagen than type III collagen ($p<0.001$ for medium, $p<0.01$ for cells).

Total type I and type III collagen decreased but remained higher than the normal values ($p<0,0002$ for type I collagen $p<0,0001$ for type III collagen).

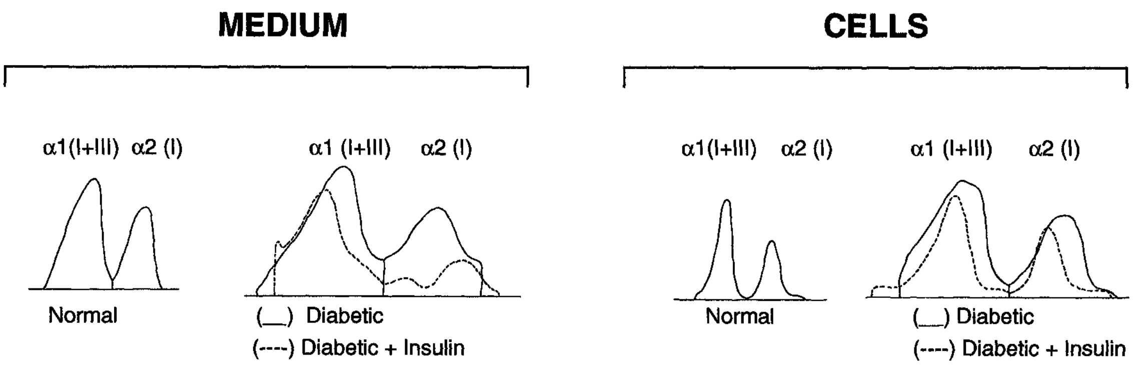

FIGURE 7 Qualitative densitometric analysis of α1 (I+III) and α2 (I) collagenous of cultured SMCs in the three experimental conditions studied (N, D, D+1). The collagen samples were analysed after reduction with 0.25% β mercaptoethanol by electrophoresis on 10% SDS polyacrylamide gel. Gels were stained with Coomassie blue and discoloured in 10% acetic acid.

**Immunological Procedures**

Examination of the medium and cells by the immunoblott procedure demonstrated in Figure 6 that cultures of SMCs synthesised type I collagen which was secreted in the medium. In fact, the cell matrix and the medium contained both α1 and α2 chains. In diabetic state, the immunological identification of type I collagen in medium and cell-matrix showed a pro-nounced increase of α1 and α2 chains in the two compartments as observed in Figure 6. Otherwise, the antiserum used is known to react favourably with both chains.

Before the biosynthesis study procedure, a densitometric analysis was made SDS-PAGE gel. The profiles of α chains of collagen types, obtained from medium and cells are shown in Figure 7. They showed more modifications in diabetic state and under insulin effect.

### DISCUSSION

According to the works of Marquié *et al.* [16,23] and Lahfa *et al.*, [24] *Psammomys obesus* under hypercaloric diet during one month developed obesity with hyperinsulinemia and after three months developed diabetic syndrome characterised by hyperglycemia and elevateddisturb lipid concentration.

The cultured SMCs of diabetic *Psammomys obesus* presented striking changes. The morphometric method revealed an increase of cellular and nuclear great axes compared with normal cells. The number of nucleoli was also increased by 1.6. These changes could be in relation to the functional state of the cells which develop many organelles in the synthetic state such as ribosome, ergastoplasm, Golgi, *etc.* [25] Simons and Vanderbroeck [26] have noted these morphometric changes in aged cells [26] and the early ageing characterised the diabetic angiopathy. [27]

In diabetic state, collagen biosynthesis is marked by a statistical increase of the labelled proline pool in ECC (x5.6) and ICC (x9.3), corresponding to the anarchic synthesis and disorganisation of the ECM during the atherosclerotic process development. [12]

The newly synthesised collagen that constitutes the major ECM component,[28] marks the aggravation of the cellular alteration due to diabetes and causes fibrosis state. [29,30,31] In fact, the fibrosis of the aortic wall would be due to the presence of collagen, [32] which is dominant in the proliferative intima. [33]

Our qualitative and quantitative study on the phenotypic expression of collagen has shown that cultured SMCs of the normal and diabetic *Psammomys obesus* synthesise more type I than type III collagen, in accord with the results of

Layman *et al.* [34] in SMCs of adult humans, pigs and rabbits. Carey *et al.* [35] and Lawrence *et al.* [36] have shown that SMC produced essentially collagen I and III. Ang *et al.* [37] have observed an activation of mRNA coding α1 (I) and α1 (III) chains of procollagen in rabbit synthetic SMCs.

Compared with control, the diabetic state induced a large increase in type I and type III collagen biosynthesis and secretion. In addition, comparatively to collagen III, collagen I appeared more important in the medium (x10.8 *vs* x9.3) than in the cells (x13.6 *vs* x11.6). Robert [29] indicated that the newly synthesised collagen in the atherosclerotic lesions is due to type I collagen. Karim *et al.* [33] have noted equally an increase of mRNA coding for α1 (I) α1 (III) chains after 30 days of experimental angioplasty. Katsuda *et al.* [32] have shown the coexistence of the two phenotypes in the intimal lesions, but type I collagen would stimulate the SMC migration. [38] During the development of the atherosclerotic process, many authors showed that collagen molecules are also subject to qualitative alterations.

These alterations would correspond to the progressive insolubility [39] and to the nonenzymatic glycosylation [40] which accelerates the macroangiopathic progression. [1,6] These processes would aggravate with diabetes. [41] However, the qualitative alterations of collagen have no influence on the immunological identification of α1 and α2 chains of type I collagen during diabetes.

In our study, the low dose insulin (4 mU/ml) at short term (48h) presented a therapeutic effect. Insulin promptly reduced all studied morphological and biochemical parameters of the SMCs of diabetic *Psammomys obesus*. In fact, the ECM synthesis of type I collagen especially the elevated ratio type I / type III collagen which was observed in diabetic *Psammomys obesus* SMCs, pronouncedly decreased. Thus, our results suggest that insulin at low dose induced inhibitory effects as compared with high doses studied by Kjelstöm and Mainquist [42] on fibroblasts of diabetic patients according with Stout [43] and Sowers. [44] They observed that high doses used are comparable to hyper-insulinemia and accelerated the atherosclerotic process in obese and diabetic patients. In addition, we have observed that SMCs activities of diabetic *Psammomys obesus* decreased under insulin effect but remain more important than the controls.

### *Acknowledgments*

The authors express their thanks to Mr. Johnson for his help in the styling of the manuscript.

### *References*

[1] Ross, R., (1986). The pathogenesis of atherosclerosis. N. *Engl. J. Med.*, **314**, 488–500.

[2] Campbell, G. R. and Chamley – Campbell, J. H. (1981). Smooth muscle in atherosclerosis. *Pathology*, **13**, 424–39

[3] Guyton, J. R. (1994). The arterial wall and the atherosclerotic lesion. *Curr. Opin. Lipidol.*, **5**, 376–81.

[4] Vogel, R. A. (1997). Coronary risk factors, endothelial function and atherosclerosis: a review. *Clin. Cardial.*, **20**, 426–32

[5] Nayler, W. G. (1994). Therapeutic approches to the control of coronary atherosclerosis. *Basic-Res. Cardiol.*, **89**, suppl 1, 137–43.

[6] Sanders, M. (1994). Molecular and cellular concepts in atherosclerosis. *Pharmacol. Ther.*, **61**, 109–53.

[7] Thyberg, J., Hedin, U., Sjound, M., Palmerg, L. and Bottger, A. (1990). Regulation of differentiated properties and proliferation of arterial smooth muscle cells. *Arteriosclerosis.*, **86**, 123–137.

[8] Bourdillon, M. C., Dussere, E., Covacho, C. and Berthezene, F. (1991). Modulation des cellules musculaires arterielles en culture et mouvements de cholesterol. *Annales d'Endocrinologie*, **52**, 464–466.

[9] Browns, M. S. and Goldstein, J. L. (1984). How LDL receptors influence cholesterol and atherosclerosis. *Sci. Am.*, 251–52.

[10] Leuteneger, M. and Bertini, E. (1995). Diabetes mellitus and atherosclerosis. Physiopathology of diabetic macroangiopathy. *Rev. Med. Interne*, **16**, 31–42.

[11] Schneider, D. J and Sobel, B. E. (1997). Determinants of vascular disease in patients with type II diabetes and their therapeutic implications. *Clin. Cardiol.*, **20**, 433–40

[12] Robert, L. and Labat-Robert, J. (1989). Morphogenesis, aging and repair of the connective tissues. *Facial Plastic Surgery*, **6**, 1–7

[13] Robert, L. and Peterszgi, G. (1998). Aging and matrix biology. *Path Biol.*, **46**, 491–495.

[14] Marquié, G., Petkov, P. and Duhault, J. (1980). Diabetic syndrome in sand rat (*Psammomys obesus*) with special reference to the pancreas. *Atherosclerosis*, **47**, 7–17.

[15] Daly, M. and Daly, S. (1973). On the ecology of *Psammomys obsesus* (Rodentia gerbillidae) in the wadi saoura Algeria. *Mammalia*, **37**, 546–561.
[16] Marquié, G., Duhault, J. and Jacotot, B. (1984). Diabetes Mellitus in Sand Rats (*Psammomys obesus*). Metabolic Pattern During Development of the Diabetic Syndrome. *Diabetes.*, **33**, 438–443.
[17] Ross, R. (1971). The smooth muscle cell. II. Growth of smooth muscle in culture and formation of elastic fibers. *The Journal of Cell Biology*, **50**, 172–186.
[18] Bourdillon, M. C., Boissel, J. P. and Crouzet, B. (1977). Proliferation of primary cultures from rat aortic media. Effects of hyperlipidemic serum. *Prog. Biochem. Pharmacol.*, **13**, 103–110.
[19] Peterkofsky, B., Chojkier, M. and Batman, J. (1982). Determination of collagen synthesis in tissue and cell culture systems. In: *Immunocytochemistry of the Extracellular Matrix*, Ed. Furthmayr M. D. H., **2**, CRC Press. 19–48.
[20] Wiestner, M., Rohde, H., Helle, O., Krief, T., Timpl, R. and Muller, P. K. (1982). Low rate of procollagen conversion in dermatosparatic sheep fibroblasts is parallel by increased synthesis of type I and type III collagen. *EMBO. J.*, **1**, 513–516.
[21] Laemmli, U. K. (1970). Cleavage of structural proteins during the assembly of the head of bacteriophage T4. *Nature*, **227**, 680–685.
[22] Towbin, H., Stahelin, T. and Gordon, J. (1979). Electrophoretic transfer of protein from polyacrylamide gels to nitrocellulose sheets; procedure and some explications. *Biochem.*, **76**, 4350–4354.
[23] Marquié, G., Dufour, P., Semmar, S., Michoudet, C., Koceir, E. L., Ahmed Bey, D., Bouguerra, S. and Messaoudi, S. (1982). Diabéte sucré chez le rat des sables (*Psammomys obesus*). *Bull. Soc. Hist. Nat. Afr. Nord. Alger.* **70**, 65–78.
[24] Lahfa, F. B., Dahmani, Y., Troutaud, D. and Deschaux, P. (1995). Nutritional influences on *in vitro* splenic lymphocyte proliferation in *Psammomys Obesus*. *Cell. and Mol. Biol. Res.*, **41**, 387–390.
[25] Godeau, G. (1990). La cellule musculaire lisse. *Revue de la veine et du lymphatique*, **4**, 8–11.
[26] Simons, J. and VanderBroeck, C. (1970). Comparison of ageing *in vitro* and ageing *in vivo* by means of cell size analysis using a coulter counter. *Gerontol*, **16**, 340–351.
[27] Mossaz, A. and Assal. J. P. (1987). Aspects physiopathologiques et cliniques de l'angiopathie diabétique. In: *paroi artérielle et diabéte*, Camilleri, J. P., Berry, C. L., Fiessinger, J. N. and Bariety, J. (1987). Ed., Flammarion.
[28] Nagler, A., Miao, H. O., Aingorm, H., Pines, M., Genina, O. and Vlodowsky, I. (1997). Inhibition of collagen synthesis, smooth muscle cell proliferation and injury induced intimal hyperplasia by halofuginone. *Arterioscler. Thromb. Vasc. Biol.*, **17**, 194–202.
[29] Robert, L. (1993). Pathogénie de l'athérosclérose. In: «athérosclérose». Chap. 3, 49–89.
[30] Macleod, D. C., Straws, B. H., De Jong, M., Escaned, J., Umans, V. A., Van-Suyelen, R. J., Verkek, A., De Feyter, P. J. and Serruys, P. W. (1994). Proliferation and extracellular matrix synthesis of smooth muscle cells cultured from human coronary atherosclerotic and restenotic lesions. *J. Am. Coll. Cardiol.*, **23**, 59–65.
[31] Ferreira-Montero, I. J., Ferreira-Aguar, A. I. and Casasnovas-Lenguas, J. A. (1995). The pathogenesis of the evolution of the atheroma plaque. *Rev. Esp. Cardiol.*, **48**, 13–22.
[32] Katsuda, S., Okada, Y., Minamoto, T., Oda, Y., Matsui, Y. and Nakanishit, T. (1992). Collagens in humam atherosclerosis, immunochemical análysis using collagen type specific antibody. *Arterioscler Tromb.*, **12**, 494–502.
[33] Karim, M. A., Miller, D. D., Farrar, M. A., Eleftheriades, E., Reddy, B. H., Brehand, C. M. and Samarel, A. M. (1995). Histomorphometric and biochemical correlates of arterial procollagen gene expression during vascular repairs after experimental angioplasty. *Circulation.*, **91**, 2049–57.
[34] Layman, D. L., Epstein, E. H., Dodson, R. F. and Titus, J. L. (1977). Biosynthesis of type I and type III collagens by cultured smooth muscle cells from human aorta. *Natl. Acad. Sci. USA*, **74**, 671–675.
[35] Carey, D. J. (1991). Control of growth and differentiation of vascular cells by extracellular matrix proteins. *Ann. Rev. Physiol.*, **53**, 161–77.
[36] Lawrence, R., Hartmann, D. J. and Sonenshein, G. E. (1994). Transforming growth factor beta 1 stimulating type V collagen expression in bovine vascular smooth muscle cells. *J. Biol. Chem.*, **269**, 9603–9.
[37] Ang, A. H., Tachas, G., Campbell, J. H., Batman, J. F. and Campbell, G. R. (1990). Collagen synthesis by cultured rabbit aortic smooth muscle cells. *Alteration with Phenotype. Biochem. J. 265*, **2**, 461–469.
[38] Powel, R. J., Hydowski, J., Frank O., Bhargava, J. and Sampio, B. E. (1997). Endothelial cell effect on smooth muscle cell collagen synthesis. *J. Surg. Res.*, **69**, 113–8.
[39] Sakata, N., Meng, J., Jimi, S., Segawa, M. and Takebayaski, S. (1995). Aging of aorta and atherosclerosis, Role of nonenzymatic glycation of collagen. *Nippon. Ronen. Igakhair. Zazski*, **32**, 336–43.
[40] Brownlee, M., Cerami, A. and Vlassara, H. (1988). Advanced glycosylation end-products in tissue and the biochemical basis of diabetic complications. *N. Engl. J. Med.*, **318**, 1315–1321.
[41] Taniguchi, N., Kaneto, H., Asahi, M., Takahashi, M., Wenyi, C., Higashiyama, S., Fujii, J., Suzuki, K. and Kayanoki, Y. (1996). *Involvea of Glycation and Oxidative Stress in Diabetic Macroangiopathy Diabetes.*, **45**, 581–583.
[42] Kjellström, T. and Malmquist, J. (1984). Insulin effects of collagen and protein production in cultured human skin fibroblasts from diabetic and non diabetic subjects. *Horm. Metabol. Res.*, **16**, 168–171.
[43] Stout, R. W. (1979). Diabetes and atherosclerosis, the role of insulin. *Diabetologia*, **16**, 141–150.
[44] Sowers J. (1992). Insulin resistance, hyperinsulinemia, dyslipidemia, hypertension and accelered atherosclerosis. *J. Clin. Pharmacol.*, **32**, 529–535.

